# Construyo e validado de jogo educativo sobre sexualidade para adolescentes*

**DOI:** 10.15649/cuidarte.2354

**Published:** 2023-05-27

**Authors:** Nadyelle Elias Santos-Alencar, Maria Aparecida Oliveira-Pinto, Nicácio Torres-Leite, Claudia Maria Vieira-da-Silva

**Affiliations:** 1 . Instituto Federal de Educado, Ciencia e Tecnologia do Maranháo, Pedreiras, MA, Brasil. Universidade Federal do Ceará, Fortaleza, CE, Brasil. Email: nadyelle-elias@hotmail.com Universidade Federal do Ceará Universidade Federal do Ceará Fortaleza CE Brazil nadyelle-elias@hotmail.com; 2 . Instituto Federal de Educado, Ciencia e Tecnologia do Maranháo, Pedreiras, MA, Brasil. Email: mariapinto@acad.ifma.edu.br Instituto Federal de Educação Ciência e Tecnologia do Maranhão Instituto Federal de Educado, Ciencia e Tecnologia do Maranháo Pedreiras MA Brazil mariapinto@acad.ifma.edu.br; 3 . Instituto Federal de Educado, Ciencia e Tecnologia do Maranháo, Pedreiras, MA, Brasil. Email: nicacio.torres@acad.ifma.edu.br Instituto Federal de Educação Ciência e Tecnologia do Maranhão Instituto Federal de Educado, Ciencia e Tecnologia do Maranháo Pedreiras MA Brazil nicacio.torres@acad.ifma.edu.br; 4 . Instituto Federal de Educado, Ciencia e Tecnologia do Maranháo, Pedreiras, MA, Brasil. Universidade Estadual do Ceará, Fortaleza, CE, Brasil. Email: claudia.vieira@ifma.edu.br Universidade Estadual do Ceará Universidade Estadual do Ceará Fortaleza CE Brazil claudia.vieira@ifma.edu.br

**Keywords:** Tecnologia Educacional, Sexualidade, Adolescente, Educational Technology, Sexuality, Adolescent, Tecnología Educacional, Sexualidad, Adolescente

## Abstract

**Introdujo:**

a educagáo em saúde de adolescentes com foco na sexualidade é favorecida pelo emprego de tecnologias cuidativo- educacionais.

**Objetivo:**

construir e validar uma tecnologia educacional, do tipo jogo de tabuleiro, para mediar a discussáo de tópicos referentes a sexualidade com a populado adolescente.

**Materiais e métodos:**

estudo metodológico delineado em tres etapas, a saber: 1°) delimitado do conteúdo a ser abordado, 2°) elaborado do protótipo do jogo, 3°) validado do conteúdo e aparencia por juízes especialistas.

**Resultados e discussao:**

"Match" é um jogo de tabuleiro, com perguntas temáticas relacionados a sexualidade que direcionam o jogador do início ao fim do trajeto. Deu-se enfase a incorporado de elementos da gamificagáo, tais como, sorte, estratégia, competido e cooperado. O processo de validado obteve éxito em todas as categorias avaliadas, o jogo foi considerado relevante e adequado as atividades de educado em saúde.

**Conclusdes:**

O jogo elaborado encontra-se validado e disponível a comunidade científica e profissional, e deverá ter os seus efeitos mensurados nas próximas etapas da pesquisa. Além disso, uma versáo digital da ferramenta deve ser construída para elevar o seu alcance.

## Abstract (en)

**Introduction:**

health education for adolescents with a focus on sexuality is favored using care- educational technologies.

**Objective:**

to build and validate an educational technology, like a board game, to mediate the discussion of topics related to sexuality with the adolescent population.

**Materials and methods:**

methodological study outlined in three stages, namely: 1st) delimitation of the content to be addressed, 2nd) creation of the game prototype, 3rd) validation of the content and appearance by expert judges.

**Results and discussion:**

“Match” is a board game, with thematic questions related to sexuality that direct the player from the beginning to the end of the path. Emphasis was placed on the incorporation of gamification elements, such as luck, strategy, competition, and cooperation. The validation process was successful in all evaluated categories, the game was considered relevant and suitable for health education activities.

**Conclusions:**

The developed game is validated and available to the scientific and professional community, and its effects should be measured in the next stages of the research. In addition, a digital version of the tool must be built to increase its reach.

## Resumen

**Introducción:**

la educación en salud de los adolescentes con enfoque en la sexualidad se ve favorecida por el uso de tecnologías cuidado - educativas.

**Objetivo:**

construir y validar una tecnología educativa, a modo de juego de mesa, para mediar en la discusión de temas relacionados a la sexualidad con la población adolescente.

**Materiales y métodos:**

estudio metodológico planteado en tres etapas, a saber: 1^a^) delimitación del contenido a abordar, 2^a^) creación del prototipo del juego, 3^a^) validación del contenido y apariencia por jueces expertos.

**Resultados y discusión:**

“Match” es un juego de mesa, con preguntas temáticas relacionadas con la sexualidad que dirigen al jugador desde el principio hasta el final del viaje. Se hizo hincapié en la incorporación de elementos de gamificación, como la suerte, la estrategia, la competencia y la cooperación. El proceso de validación fue exitoso en todas las categorías evaluadas, el juego fue considerado relevante y adecuado para actividades de educación en salud.

**Conclusiones:**

el juego desarrollado está validado y disponible para la comunidad científica y profesional, y sus efectos deben medirse en las próximas etapas de la investigación. Además, se debe construir una versión digital de la herramienta para aumentar su alcance.

## Introdujo

A sexualidade, enquanto construyo evolutiva da espécie humana, resulta de um processo social, histórico e cultural únicos. Para além das especificidades biológicas com fins reprodutivos, a sexualidade revela-se nas entrelinhas das relagóes humanas em busca dos prazeres do corpo, sendo, por tal razao, frequentemente associada a perversao, pecado ou atentado ao pudor. O moralismo e os tabus relacionados ao assunto dificultam a sua plena discussao, corroboram para o nao entendimento de aspectos fundamentais da vida sexual humana e inibem o exercício saudável da sexualidade^1^.

Na adolescencia, fase de transigao dos 10 aos 19 anos de idade, o amadurecimento sexual marca o início de intensas transformagóes de ordem física, psíquica e social^2^. Nesse período de descobertas, ao passo em que os riscos e as vulnerabilidades se agugam, há poucas oportunidades para a conscientizagao sobre saúde sexual e reprodutiva^3^. A carencia de espagos para o debate impele a busca desordenada por informagóes, por vezes distorcidas ou inadequadas, sobretudo por meio da internet, capazes de impactar negativamente os comportamentos adolescentes^4^.

Ressalta-se que por se tratar de um grupo populacional marcado por questóes de saúde específicas, os adolescentes carecem de políticas públicas que atendam as demandas próprias da fase. Nesse contexto, as agóes de educagao em saúde devem orientar a mudanga de comportamento, a redugao das vulnerabilidades e a minimizagao dos riscos, por meio de estratégias inovadoras e atrativas, que permitam maior aceitagao e eficácia, a exemplo das tecnologias cuidativo-educacionais^5^.

No rol de tecnologias para fins de saúde, um estudo de revisao que mapeou as publicagóes ocorridas entre os anos de 1985 e 2018, revelou concentragao nos continentes americano e europeu e crescimento acentuado no número de publicagóes a partir de 2011. Assinala-se que, dentre as temáticas-base, dos 161 artigos incluídos, 65 eram dedicados a populagao de até 18 anos de idade e apenas seis tinham como foco a sexualidade^6^.

De modo mais específico, sobre jogos educativos com enfase na sexualidade, publicados até julho de 2013, uma revisao sistemática destacou a carencia de opgóes tecnológicas em países menos desenvolvidos e a concentragao massiva de estudos norte-americanos, seis dos sete estudos identificados^7^. Na literatura recente, evidencias apontam que os jogos educativos sobre sexualidade sao bem aceitos pela populagao adolescente, sendo também bem vistos pela família^4^. Além disso, auxiliam a aquisigao de conhecimento e promovem a mudanga de atitudes relacionadas a sexualidade^8^^,^^9^. Por outro lado, salientam a necessidade de aprofundamento investigativo acerca do efeito dos jogos, com foco em estudos metodológicos mais robustos^7^^,^^10^.

Com base no exposto, objetiva-se a construgao e validagao de uma tecnologia educacional, do tipo jogo de tabuleiro, para mediar a discussao da sexualidade com a populagao adolescente.

## Materiais e Método

### Tipo de Estudo

Estudo metodológico sobre a construgao e validagao de uma tecnologia cuidativo-educacional, do tipo jogo de tabuleiro, sobre sexualidade na adolescencia.

### Local de Estudo

O estudo foi desenvolvido por pesquisadores vinculados ao Instituto Federal do Maranhao, Campus Pedreiras; e ao Grupo de Estudos e Pesquisas em Inovagáo, Saúde e Sociedade - GEPISS, certificado pelo Conselho Nacional de Desenvolvimento Científico e Tecnológico (CNPQ) em 2020.

### Participantes do Estudo

A equipe de desenvolvimento da ferramenta envolveu duas pesquisadoras da área da saúde, uma enfermeira e uma profissional da educagáo física, e dois estudantes adolescentes.

Em relagáo ao grupo de especialistas, responsáveis pela validagáo da ferramenta, foi composto por nove profissionais com expertise na temática-foco.

### Procedimentos metodológicos para a elaborado e validado do jogo educativo

A elaborado do jogo seguiu tres etapas metodológicas. Inicialmente, foi delimitado o conteúdo da tecnologia a ser construída. Por entender a amplitude da temática sexualidade, procurou-se demarcar a abordagem para além das questóes biologicistas, dando lugar as vivencias adolescentes. Para isso, foi realizado um levantamento da literatura, a partir dos manuais, cartilhas e outros documentos oficiais publicados por Institutes nacionais e internacionais, com notória representatividade acerca da temática, tais como: Ministério da Saúde, Organizado Panamericana da Saúde e Organizado das Nagóes Unidas.

Na segunda etapa, foi elaborado o protótipo do jogo, com auxílio de uma ferramenta de design online e gratuita. A construgáo da tecnologia educativa deu-se através da adaptado de do jogo de tabuleiro “Cuca Legal", que envolve um quiz de perguntas e respostas sobre diferentes temas e direciona o jogador do inicio ao fim de um trajeto^11^. Nessa fase, foram definidos a apresentado identitária do jogo (nome e design) e o conteúdo (perguntas, regras do jogo e manual do aplicador).

Na última etapa, ocorreu a validado do jogo por especialistas, segundo o conteúdo (objetivo, conteúdo e relevancia) e a aparéncia (design, clareza e organizado). O formulário de validado foi adaptado do proposto por Sousa e colaboradores^12^. Os especialistas julgaram os itens com as seguintes respostas: 0-discordo totalmente; 1-discordo parcialmente; 2-náo discordo, nem concordo; 3-concordo parcialmente; 4-concordo totalmente. Além disso, os profissionais fizeram comentários e sugestóes sobre as dimensóes avaliadas.

O comité foi composto por nove especialistas^5^. A escolha dos especialistas teve como critérios a formado e a experiencia profissional. Foram incluídos profissionais com escore igual ou superior a cinco pontos, segundo os critérios, somados cumulativamente: doutorado na área da saúde (4 pontos), mestrado na área da saúde (3 pontos), doutorado/mestrado sobre saúde do adolescente, educado em saúde, tecnologia educativa e/ou validado (2 pontos), atuagáo mínima de um ano em saúde do adolescente ou educado em saúde (2 pontos), cursos de capacitagáo/especializagáo em saúde do adolescente ou educado em saúde (2 pontos).

Os juízes foram contactados por email e convidados a participar da validado. Aqueles que aceitaram, receberam uma versáo digitalizada do jogo (conteúdo e regras para uso), juntamente com o termo de consentimento livre e esclarecido, o formulário para caracterizado do perfil dos juízes e o instrumento para validado da ferramenta, no prazo de 21 dias.

### Procedimentos de análise e tratamento dos dados

Para determinar a concordancia entre as respostas dos juízes foi calculado o Índice de Validade de Conteúdo (IVC) para cada item avaliado (número de respostas “3” ou “4” / número total de respostas), com ponto de corte de 0,8. Os dados foram organizados e processados por meio do Microsoft Excel, e armazenados no Mendeley Data^13^.

### Aspectos éticos

O projeto foi submetido ao Comité de Ética em Pesquisa, respeitando os preceitos da Resolugáo 466/2012 do Conselho Nacional de Saúde, e obteve aprovagáo pelo Comité do Instituto Federal do Piauí, parecer n° 4.464.169

## Resultados

### A tecnologia cuidativo-educacional

**Figura 1:**
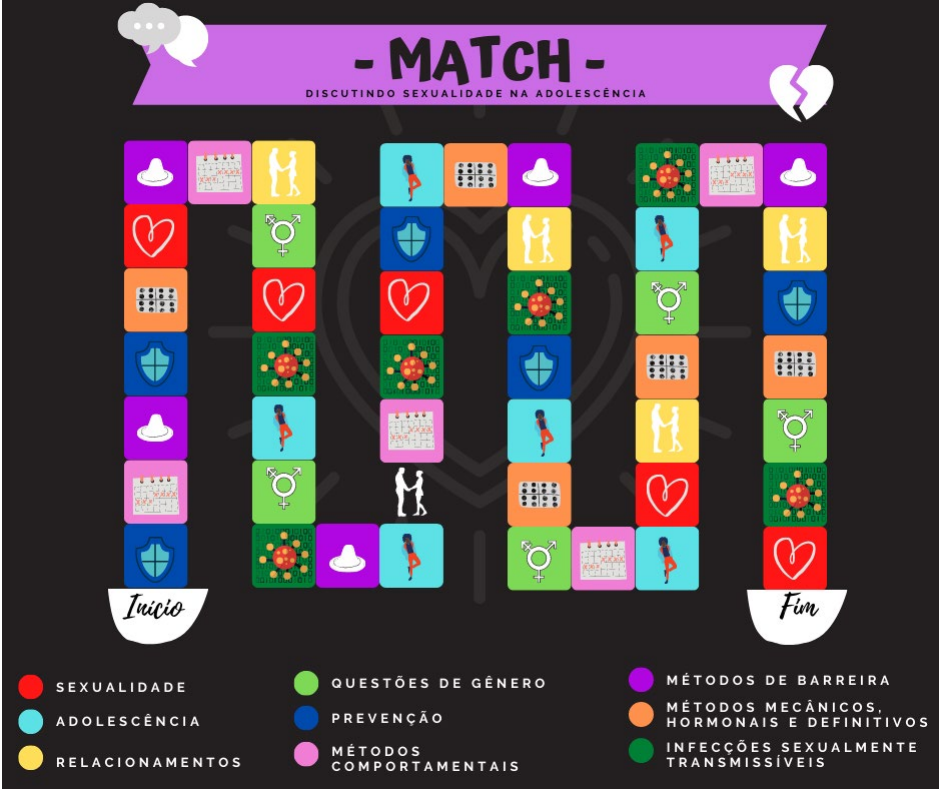
Tabuleiro do jogo “Match - Discutindo Sexualidade na Adolescencia". Pedreiras, Maranhao, Brasil, 2021.

O jogo de tabuleiro recebeu o nome “Match - Discutindo Sexualidade na Adolescéncia”. A seguir seráo detalhados os componentes e as regras que orientam o uso da tecnologia construida. Em relagáo aos seus componentes, contém: um tabuleiro, um guia com as regras, seis peóes, um dado e 40 cartas (cada carta possui nove perguntas temáticas). Os temas trabalhados sáo representados por cores e símbolos e abordam conteúdos relacionados a educagáo sexual na adolescéncia, sáo eles: adolescéncia, sexualidade, relacionamentos, questóes de género, prevengáo, métodos contraceptivos e infecgóes sexualmente transmissíveis (1ST) (Figura 1).

Em relagáo as perguntas temáticas, optou-se por incluir questóes abertas, de múltipla escolha, verdadeiro ou falso, além de situagóes-problema.

Para a jogabilidade, sao necessários de dois a seis participantes (ou grupos de participantes). A aplicagáo deve ser orientada preferencialmente por um profissional da saúde. Foi elaborado um manual de apoio ao profissional que contém todas as perguntas e respostas do jogo, fornece dicas para leitura complementar e fotos de apoio para facilitar a discussáo das temáticas durante o jogo.

A dinámica do jogo Match envolve perguntas e respostas, no qual o objetivo do jogador é acertar as perguntas temáticas, percorrer o trajeto, e chegar primeiro ao final do tabuleiro. O progresso no jogo é percebido quando o jogador acerta o maior número de perguntas, avanza as casas do tabuleiro e mantém-se a frente dos demais competidores.

No que tange a mecánica, o jogador que inicia a partida langa o dado e, de acordo com o número obtido, alcangará uma casa temática do tabuleiro. Em seguida, deve buscar uma carta no monte e responder a questáo correspondente. Caso erre, deverá retornar a posigáo anterior. Se acertar, permanece na casa e passa a vez para o jogador seguinte. Assim acontece o jogo, até que um dos jogadores alcance o final.

Sobre algumas estratégias de gamificagáo incorporadas ao jogo, destacam-se a sorte, a estratégia, a competigáo e a cooperagáo. A sorte acontece essencialmente através do uso do dado que define quantas casas devem ser percorridas. A estratégia é evidenciada pela regra do “náo sei”, na qual o jogador pode abster-se de responder a questáo quando náo sentir seguranga, em apenas duas oportunidades durante a partida, estimulando a tomada de decisáo do jogador.

Além disso, a relagáo dos jogadores é evidenciada de duas formas: a competigáo se dá naturalmente pelo desejo de ganhar chegando primeiro a última casa do jogo e a cooperagáo ocorre quando o jogo é disputado entre grupos de jogadores, e a resposta a pergunta deve ser definida em consenso pelos integrantes do grupo.

### A validagáo da tecnología cuidativo-educacional

Nove juízes especialistas participaram do processo de validagáo, seis deles do sexo feminino, com idade entre 28 e 40 anos e renda mensal maior que 4 Salários Mínimos (SM). Todos eram enfermeiros, com formagáo específica em diversas áreas, tais como saúde da família, saúde da mulher, epidemiologia e saúde mental (Tabela 1).

**Tabela 1:** Caracterizagáo dos juízes especialistas. Pedreiras, Maranháo, Brasil, 2021.

	Sexo	Idade	RMM	Profissao	Formagao	Tempo de atuagao (saúde do adolescente)
Juiz 1	Feminino	31	>6 a 8 SM	Enfermeiro	Especializagao em Saúde da Família e Enfermagem do trabalho	6
Juiz 2	Masculino	30	>4 a 6 SM	Enfermeiro	Especializagao em Atengao Básica e Saúde da Família	1
Juiz 3	Feminino	29	> 10 SM	Enfermeiro	Especializagao	6
Juiz 4	Feminino	28	>4 a 6 SM	Enfermeiro	Especializagao em Enfermagem do Trabalho e Saúde Mental	6
	Sexo	Idade	RMM	Profissao	Formado	Tempo de atuado (saúde do adolescente)
Juiz 5	Masculino	38	>6 a 8 SM	Enfermeiro	Especializado em Saúde da Mulher Negra	6
Juiz 6	Masculino	33	>6 a 8 SM	Enfermeiro	Mestrado em Epidemiologia	5
Juiz 7	Feminino	31	> 10 SM	Enfermeiro	Especializado em Enfermagem obstétrica e neonatal	6
Juiz 8	Feminino	40	>4 a 6 SM	Enfermeiro	Residencia em Enfermagem Obstétrica	6
Juiz 9	Feminino	34	>6 a 8 SM	Enfermeiro	Especializado em Enfermagem do trabalho	6

*Legenda: RMM - renda média mensal; SM - salário mínimo (1SM= 1.045,00).*

A Tabela 2 apresenta a validagáo do jogo Match quanto as dimensóes: objetivos, conteúdo e relevancia. Além da distribuigáo dos profissionais segundo a resposta fornecida, foi calculado o IVC, geral e por item, para avaliar a concordancia entre eles. Em relagáo as tres dimensóes mencionadas, obteve-se IVC máximo (1,0).

**Tabela 2:** Avaliaáo dos objetivos, conteúdo e relevancia do jogo Match segundo o Índice de Validade de Conteúdo. Pedreiras, Maranháo, Brasil, 2021.

	DT DP NDNC CP	CT	IVC
Objetivos			
O jogo atende aos objetivos propostos?	- - - -	9	1,0
O jogo permite a discussao sobre temas relacionados a		9	1,0
sexualidade na adolescencia?			
O jogo é adequado para a utilizado por profissionais		9	1,0
que trabalham com adolescentes? Conteúdo			
O conteúdo é adequado a temática?	- - - -	9	1,0
O conteúdo é adequado ao público?	- - - 1	8	1,0
O conteúdo é completo e abrangente?	- - - 1	8	1,0
As informaóes apresentadas sao corretas?	- - - -	9	1,0
Relevancia			
O jogo favorece a educado em saúde de adolescentes?	- - - -	9	1,0
O jogo é relevante para a prática profissional?	- - - 2	7	1,0
O jogo contribui para a construao de conhecimentos	- - - -	9	1,0
sobre a temática?			
Geral	- - - 4	86	1,0

*Legenda: DT- discordó totalmente, DP - discordó parcialmente, NDNC - náo discordó, nem concordo, CP - concordo parcialmente, CT- concordo totalmente, IVC - índice de validade de conteúdo.*

A validagáo da clareza, organizado e design está demonstrada na Tabela 3 Nenhum dos itens gerou discordancia entre os juízes, entretanto, aqueles relacionados ao design tiveram um IVC abaixo das demais dimensóes avaliadas. Vale ressaltar que todos os itens tiveram avaliagáo satisfatória, superando o valor mínimo de concordancia de 0,8.

**Tabela 3:** Avaliaáo da clareza, organizado e design do jogo Match segundo o índice de Validade de Conteúdo. Pedreiras, Maranháo, Brasil, 2021.

	DT DP NDNC	CP	CT	IVC
Clareza e Organizado				
As regras sao claras e de fácil entendimento?	- - -	3	6	1,0
O material utilizado está apropriado?	- - -	1	8	1,0
O conteúdo das cartas é adequado?	- - -	0	9	1,0
Design				
O design das cartas é adequado?	- - 1	-	8	0,89
A fonte dos textos (títulos e corpo do texto) é adequada?	- - 1	1	7	0,89
A redado e o vocabulário sao acessíveis ao público-alvo?	- - -	1	8	1,0
Geral	- - 2	6	46	0,96

*Legenda: DT- discordó totalmente, DP - discordó parcialmente, NDNC - nao discordó, nem concordo, CP - concordo parcialmente, CT- concordo totalmente, IVC - índice de validade de conteúdo.*

Além do julgamento dos itens, os juízes fizeram sugestóes e comentários. O juiz 9 afirmou que o jogo será de grande relevancia para estimular o interesse e a aprendizagem. Sobre o modo como a temática foi trabalhada, o juiz 7 enalteceu o conteúdo do material de apoio ao profissional e salientou que o uso de perguntas com casos-problema é uma importante estratégia para a discussáo das situagóes cotidianas com os jovens.

**Figura 2:**
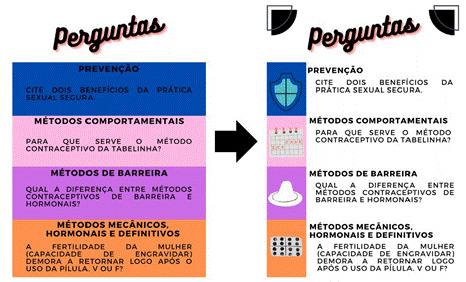
Modelo das cartas do jogo “Match - Discutindo Sexualidade na Adolescencia" com perguntas temáticas, antes e após o processo de validado. Pedreiras, Maranhao, Brasil, 2021.

Em relagáo ao design, o juiz 3 sugeriu o uso de tons mais claros nas cartas para facilitar a leitura dos textos, além do uso de vocábulos mais informais em algumas das perguntas do jogo. No mesmo sentido, o juiz 8 propós a inclusáo/substituigáo de alguns vocábulos para a melhor compreensáo pelo público-alvo, a exemplo da incorporado do termo "pílula do dia seguinte” como sinónimo de "anticoncepcional de emergencia”. Conforme as sugestóes dos juízes, o modelo das cartas foi alterado (Figura 2).

Tal como o design, os avaliadores sugeriram mudangas em algumas regras do jogo, foram elas: uso da sorte para iniciar a partida, antes o jogador mais novo iniciava; agora, inicia aquele que alcangar o maior número ao langar o dado. Também foi alterada a regra que permitia ao adolescente jogar novamente caso tirasse um número par e acertasse a questáo correspondente a casa do tabuleiro; agora, terá direito a jogar novamente, o jogador que adivinhar o número que alcangará no dado e acertar a pergunta.

## Discussao

O presente estudo apresenta e disponibiliza a comunidade científica brasileira um jogo factível de ser incorporado aos programas de educagáo em saúde, junto a populagáo adolescente. O jogo "Match - Discutindo Sexualidade na Adolescencia", validado por especialistas, inova ao abordar diversas temáticas no escopo da sexualidade, possibilitando a discussáo ampliada sobre o assunto, além de permitir diferentes experiencias a cada partida.

No processo de validagáo, o jogo revelou resultados satisfatórios em todas as categorias avaliadas. Os juízes apresentaram avaliagáo entusiasmada, inclusive apontando a relevancia e aplicabilidade prática. Ressalta-se a importancia do processo de validagáo para o refinamento, ajustes e adequagáo da ferramenta, quanto a forma e conteúdo, como etapa prévia a disponibilizagáo para uso.

A populagáo adolescente, direcionada pela curiosidade e impulsividade próprias da fase, encontra-se suscetível a comportamentos de risco que os expóem a eventos perturbadores do ciclo natural de desenvolvimento, a exemplo da elevada incidencia de IST^14^ e gravidez na adolescencia^15^. Ressalta-se que, além dos cuidados triviais com o corpo e do estímulo ao sexo seguro, defende-se que a educagáo sexual impulsione a autonomia e conscientize essa populagáo acerca dos riscos e repercussóes relacionados as vivencias sexuais. Mais especificamente, convém a ampliagáo do debate e a inclusáo de temas, tais como: relacionamentos violentos, abuso sexual, questóes de genero e identidade sexual, com foco na quebra do ciclo da violencia e prevengáo do preconceito^3^.

A despeito da relevancia, o uso de jogos para a educagáo sexual de adolescentes ainda é discreto. Uma revisáo sistemática das publicagóes sobre o tema, ocorridas até 2013, identificou apenas sete estudos, e nenhum deles tinha como foco a populagáo brasileira. De modo geral, demonstrou-se que os jogos apresentam efeitos positivos, embora haja necessidade de avaliagáo mais robusta sobre os seus impactos a longo prazo, com énfase em desenhos metodológicos rigorosos, a exemplo dos ensaios controlados e aleatorizados^7^.

As publicagóes nacionais sobre o tema sáo recentes, com destaque: "Papo Reto", jogo online que discute sexualidade e relagóes de género^16^, "DECIDIX", jogo digital sobre relagóes afetivas e sexuais^17^, "Contando bem que mal tem?", jogo de cartas que debate corporalidade, relacionamentos, IST e métodos contraceptivos^12^, e "A aventura do adolescente com Transtorno do Espectro Autista (TEA)", jogo de tabuleiro para a educagáo sexual de adolescentes com TEA^18^.

Dentre os estudos brasileiros, observa-se a caréncia de investigagóes que avaliem efetivamente o efeito das ferramentas educativas. Entretanto, resultados preliminares apontam que os jogos sáo benéficos^12^, auxiliam o ganho de conhecimento^15^, estimulam a autonomia e o compartilhamento de experiéncias^17^.

Os dados nacionais harmonizam com a realidade internacional. O jogo para computador "My Future Begins Today", por exemplo, trata sobre saúde sexual de adolescentes em áreas com baixo acesso a tecnologia na África Subsariana, e revelou que o jogo foi capaz de incitar a mudanga de atitude e promover práticas sexuais seguras^9^. Outro exemplo é o jogo de smartphone "Tumaini", como foco na prevengáo do Vírus da Imunodeficiéncia Humana (HIV) entre jovens africanos, que, ao discutir situagóes da vida real, propicia o pensamento crítico, estimula a tomada de decisáo e a solugáo de problemas, criando o senso de responsabilidade^10^.

Além disso, os jogos demonstram ser compatíveis aos interesses dos adolescentes e geram elevado engajamento^19^. O jogo "Sexpert High School" expos beneficios a educagáo sexual de adolescentes latinos e evidenciou sua elevada viabilidade e aceitabilidade^8^. Na mesma perspectiva, o jogo para tablet "My Future Family", que aborda temas relacionados a puberdade, saúde sexual e reprodutiva e contracepgáo, também demonstrou boa aceitagáo pelos adolescentes. A maioria dos alunos gostou do jogo, o recomendaria a um amigo e acredita que favoreceu o ganho de conhecimento^4^.

Contrapondo os bons resultados até aqui apresentados, o jogo de cartas "InFection Four" náo obteve avaliagáo satisfatória, apenas 27% dos participantes afirmou ter aprendido com o jogo e 26% disse náo ter aprendido nada. Cabe ressaltar que esses resultados tém relagáo com a narrativa fictícia adotada pelo jogo, uma vez que, os estudantes preferem conteúdos e abordagens que envolvam situagóes cotidianas reais^20^.

Considerando o exposto, ressalta-se que as atividades educativas, quando adequadas a realidade do adolescente, favorecem a construgáo de conhecimento e a redugáo das vulnerabilidades observadas, sendo os jogos estratégias facilitadores nesse processo^21^. Isso ocorre porque os jogos educativos apresentam valor educacional sem deixar de lado o entretenimento. Atuam como mediadores do processo de aprendizagem, permitem a discussáo de temas tabus e promovem experiéncias ricas e engajadoras, demonstrando adequagáo as agóes de promogáo da saúde^19^.

Náo obstante, é essencial reconhecer as limitagóes do presente estudo e indicar as próximas etapas a serem empregadas. Apesar de estar validado quanto ao conteúdo e aparéncia, a avaliagáo do efeito do jogo no conhecimento, atitudes e intengóes dos adolescentes precisa ser foco de futuras investigagóes. Sugere-se ainda que a avaliagáo ocorra com amostras representativas, envolvendo ambos os sexos, faixa etária abrangente e características socioculturais diversas. Outro direcionamento deve ser a construyo de uma versáo digital do jogo, corroborando para a maior acessibilidade em relagáo ao tempo e espago.

## Conclusoes

O presente estudo elaborou e validou um jogo educativo, do tipo tabuleiro, intitulado "Match - Discutindo Sexualidade na Adolescencia". Integra um grupo restrito de estudos sobre a temática na realidade brasileira, sendo uma opgáo viável e validada, em condigóes adequadas para o uso enquanto ferramenta educacional.

Segundo os juízes, o jogo demonstra pertinencia prática, conteúdo abrangente e está adequado a populagáo-alvo, sendo relevante para a prática profissional. Em relagáo a aparencia, regras e linguagem, alguns ajustes foram feitos de modo a atender as sugestóes dos profissionais especialistas na área.

Por fim, convém ressaltar a importancia das etapas seguintes para avaliagáo do efeito do jogo no conhecimento, nas tomadas de decisóes e nas intengóes dos jovens, como também a construgáo de uma versáo digital da ferramenta, de forma a torná-la mais acessível.
